# CdS nanoparticles sensitization of Al-doped ZnO nanorod array thin film with hydrogen treatment as an ITO/FTO-free photoanode for solar water splitting

**DOI:** 10.1186/1556-276X-7-593

**Published:** 2012-10-25

**Authors:** Chih-Hsiung Hsu, Dong-Hwang Chen

**Affiliations:** 1 Department of Chemical Engineering, National Cheng Kung University, Tainan, Taiwan 701, Republic of China; 2 Research Center for Energy Technology and Strategy, National Cheng Kung University, Tainan, Taiwan 701, Republic of China

**Keywords:** Al-doped ZnO nanorod thin film, Hydrogen treatment, CdS nanoparticles, ITO/FTO-free, Photoelectrode, Solar water splitting

## Abstract

Aluminum-doped zinc oxide (AZO) nanorod array thin film with hydrogen treatment possesses the functions of transparent conducting oxide thin film and 1-D nanostructured semiconductor simultaneously. To enhance the absorption in the visible light region, it is sensitized by cadmium sulfide (CdS) nanoparticles which efficiently increase the absorption around 460 nm. The CdS nanoparticles-sensitized AZO nanorod array thin film with hydrogen treatment exhibits significantly improved photoelectrochemical property. After further heat treatment, a maximum short current density of 5.03 mA cm^−2^ is obtained under illumination. They not only are much higher than those without CdS nanoparticles sensitization and those without Al-doping and/or hydrogen treatment, but also comparable and even slightly superior to some earlier works for the CdS-sensitized zinc oxide nanorod array thin films with indium tin oxide (ITO) or fluorine-doped tin oxide (FTO) as substrates. This demonstrated successfully that the AZO nanorod array thin film with hydrogen treatment is quite suitable as an ITO/FTO-free photoanode and has great potentials in solar water splitting after sensitization by quantum dots capable of visible light absorption.

## Background

In recent years, hydrogen energy has found increased attention as a renewable and clean energy source in scientific community and government organizations [1-3]. Also, it has enormous potential to be developed as a new substitutive energy resource for solving energy crisis in the future. Among many methods for the generation of hydrogen, solar water splitting is a particularly attractive one because of the environmental friendliness and the abundance of water source [4-7]. Fujishima and Honda were the first to demonstrate the concept of water splitting in a series of experiments using titanium dioxide as a photoanode [8]. Up to date, many efforts have been done on the development of photoelectrodes to improve the efficiency of hydrogen generation [6,7,9-12].

TiO_2_ is the typical photoelectrode material most extensively examined. ZnO is a cheap and safe semiconductor. Its energy-band structure and physical properties are similar to those of TiO_2_, but it has higher electronic mobility which is favorable for electron transport. So, it has the potential as an alternate of TiO_2_ in photovoltaic or photoelectrochemical devices [13]. However, both TiO_2_ and ZnO are not photocatalytic in the visible light region. Since most of the solar frequency spectrum intensity is located in the wavelength range of 400–800 nm, the drawback of nonabsorbing ability in the visible light region significantly limited their hydrogen generation efficiency in the photoelectrochemical cells [14]. So an important strategy was focused on their doping with carbon or nitrogen [15-17], the adsorption of dyes [18,19], the deposition of quantum dots [20-26], and the use of other semiconductor metal oxides capable of visible light absorption such as WO_3_[27,28] and Fe_2_O_3_[29,30] to enhance the absorption of visible light. Furthermore, to enhance the charge-transport property by increasing the direct electron conduction, the other important strategy was the development of their 1-D nanostructures such as nanorods [24,31,32], nanowires [16,21,22,26,33], and nanotubes [25,34,35].

On the other hand, it is mentionable that photoactive materials of photoelectrodes are usually coated or grown on the transparent conducting oxide (TCO) thin film for collecting electron efficiently. The suitable TCO materials are the SnO_2_, In_2_O_3_, and ZnO-based binary semiconductor compounds and the multicomponent oxides composed of combinations of these binary compounds [36]. Since the undoped oxide thin films have lower conductivity and are unstable at a high temperature, impurity doping is usually necessary in practical use. The typical examples include the F-doped SnO_2_ (FTO), Sb-doped SnO_2_ (ATO), Sn-doped In_2_O_3_ (ITO), and Al-doped ZnO (AZO) [36]. For solar energy conversion to date, the most common TCOs in dye-sensitized solar cell (DSSC), solar water splitting, and quantum dot-sensitized solar cell (QDSSC) are ITO and FTO. However, as has been known, the rare metal indium is expensive [37]. So, for DSSC, QDSSC, and the solar-driven water splitting in a photoelectrochemical cell, FTO has become the better choice. Moreover, some SnO_2_- and In_2_O_3_-based double layers or triple layers such as TiO_2_/ITO, Nb-doped TiO_2_/ITO, FTO/ITO, SnO_2_/ITO, and TiO_2_/ATO/ITO have also been developed to improve the energy conversion efficiency [38,39].

Besides the SnO_2_- and In_2_O_3_-based TCOs, AZO is another attractive TCO material because of its nontoxicity, relative abundance, low cost, thermal stability, and durability in hydrogen plasma [36,40]. Furthermore, Lee et al. has also reported that the ZnO nanowire-based DSSC using AZO as the TCO substrate showed superior cell performance than that using FTO as the TCO substrate [41]. This revealed that AZO could be used to replace ITO or FTO as the TCO substrate in DSSCs, QDSSCs, and the photoelectrochemical cell for water splitting. In addition, it is also mentionable that some efforts have been made on the development of other ITO-, FTO-, and even TCO-free electrodes in solar cells, such as the grapheme- and poly(3,4-alkylenedioxythiophene) (PEDOT)-based electrodes [42,43].

As stated above, ZnO not only is the photoactive material of photoelectrode, but also can be used as a TCO substrate after Al-doping and hydrogen treatment. Recently, we synthesized the ZnO nanorod array thin film and demonstrated that appropriate Al-doping and hydrogen treatment could lead to the significant transparency improvement and 1,000-fold conductivity enhancement [44]. This revealed that the AZO nanorod array thin film with hydrogen treatment possessed the functions of TCO thin film and photoelectrode simultaneously. Thus, the AZO nanorod array thin film with hydrogen treatment might be used directly as an ITO- and FTO-free photoelectrode. This made the fabrication of photoelectrode simple and low-cost because the use of expensive rare metal was avoided and the pre-fabrication of an extra TCO substrate is not necessary. Although the ZnO nanowire- or nanorod-based DSSC has been reported [19], TCO substrate was still used, and the phoelectrode using AZO has showed superior cell performance than using FTO [41]. So, such an AZO nanorod-based photoelectrode without an extra TCO substrate has great potentials in DSSCs, QDSSCs, and the photoelectrochemical cell for water splitting. Based on this reason, in our more recent work, the AZO nanorod array thin film with hydrogen treatment has been demonstrated to possess good photoresponse and stability [45]. Also, a preliminary test showed that its sensitization by cadmium sulfide (CdS) nanoparticles via the chemical bath deposition method could enhance the hydrogen generation efficiency efficiently because of the significant absorption of CdS nanoparticles over a wide wavelength range in the visible light region which made them useful in the development of nanocomposite photoanodes for photoelectrochemical water splitting [20-26].

Accordingly in this work, a comprehensive study has been done to develop the CdS nanoparticles-sensitized AZO nanorod array thin film as a nanocomposite photoanode for solar water splitting without an extra TCO substrate. The effect of cycle number for the chemical bath deposition of CdS nanoparticles on the photoelectrochemical properties was studied. For comparison, the photoelectrochemical properties of CdS nanoparticles-sensitized thin films without Al-doping and/or hydrogen treatment were also examined. In addition, the effect of post-heat treatment was also examined to enhance the hydrogen generation efficiency.

## Methods

AZO nanorod array thin film was synthesized in a chemical bath according to our previous work [44]. Firstly, for the deposition of ZnO seed layer, 0.4 M zinc acetate solution was prepared by dissolving zinc acetate in 11 mL of 2-methoxyethanol via sonication for 0.5 h and mixing with 0.5 mL of monoethanolamine. Then this solution was kept in a water bath at 60°C for 1 h and aged at room temperature for another 2 days. The resulting solution was deposited on the glass substrate (0.1 mL on a square of 2.5 × 2.5 cm^2^) by a spin coater at a rate of 3,000 rpm for 30 s, and then the as-deposited thin film was dried in a furnace at 350°C for 10 min to evaporate the solvent and remove organic residuals. After repeating the spin coating and drying procedures for ten times to obtain the required thickness, the obtained thin film was put into a furnace and calcined in air at 550°C for 2 h to yield the ZnO seed layer.

For the growth of AZO nanorod array thin film on the ZnO seed layer, 15 mL of aqueous solution containing zinc nitrate (0.004 M) and aluminum nitrate (Al/Zn molar ratio/20%) was mixed with the mixture of 0.46 ml diethylenetriamine and 15 mL water. After sonication for 10 min to dissolve the precursor, the solution pH was adjusted to 11.5 with 10 M NaOH to yield the deposition solution. Then the ZnO seed layer-coated glass substrate was immersed into the deposition solution and kept in an oven at 95°C for 6 h. After cooling to room temperature naturally, the glass substrate grown with AZO nanorods was washed with water and ethanol several times to remove the organic residue and dried at 70°C in an oven for 2 h. In performing hydrogen treatment to increase the crystallinity, remove organic residual, and enhance the conductivity, the as-grown AZO nanorod array thin film was annealed in Ar/H_2_ (97/3) atmosphere at 400°C with a gauge pressure of 0.4 kg/cm^2^ for 2 h. For comparison, the thin films without Al-doping and/or hydrogen treatment were also prepared according to the above method. According to our previous work [44], the resulting AZO nanorods had an average diameter of 64.7 ± 16.8 nm and an average length of about 1.0 μm.

CdS nanoparticles were decorated on the surface of AZO nanorods via a chemical bath deposition. At first aqueous solution of 30 mL containing 0.001 M cadmium nitrate, 0.005 M thiourea, and 1 M ammonium hydroxide was prepared as the deposition solution. Secondly, the AZO nanorod array thin film with hydrogen treatment was put in the deposition solution at room temperature for 1 h and then at 60°C for another 1 h. The chemical bath deposition was repeated for the desired cycle number (1–5), and the deposition solution was refreshed for each cycle. Finally, the product was washed with ethanol for several times and then dried in the oven. For the decoration of CdS nanoparticles on the thin films without Al-doping and/or hydrogen treatment, the cycle number was fixed at 3. To make sure the formation of CdS nanoparticles, the above chemical bath deposition process was also conducted in the absence of AZO nanorod array thin film for comparison.

The surface morphology was observed by a high-resolution field emission scanning electron microscopy (JEOL SEM 6700F, JEOL Ltd., Tokyo, Japan). The transmission electron micrograph (TEM), energy dispersive X-ray (EDX) spectroscopy, and high-resolution lattice image were analyzed by a high-resolution field emission transmission electron microscopy (HRTEM, JEOL Model JEM-2100F). The crystalline structures were characterized by X-ray diffraction (XRD) analysis on a Rigaku D/max-ga X-ray diffractometer (Rigaku Corporation, Tokyo, Japan) at 40 kV with Cu K_α_ radiation (λ = 0.1542 nm). The optical absorption spectra were analyzed using a Jasco V-570 UV–VIS spectrophotometer (Jasco Inc., Easton, MD, USA). The photoluminescence spectra were measured on a Hitachi F-4500 fluorescence spectrophotometer (Hitachi Company, Hong Kong, China) with a xenon lamp as the excitation source.

The CdS nanoparticles-sensitized AZO nanorod array thin film with hydrogen treatment was fabricated as the photoelectrode by sticking copper wire on the ZnO seed layer and secured with conducting copper tap. The photoelectrode was sealed on all edges with epoxy resin to reduce leakage current. Photoelectrochemical measurement was conducted on a Zahner IM6ex electrochemical workstation (Zahner*-*Elektrik GmbH & Co. KG, Kronach, Germany) in a standard three-electrode configuration with the above photoelectrode as the working electrode, Pt wire as the counter electrode, and a BAS (West Lafayette, IN, USA), Model MF-2502 Ag/AgCl electrode as the reference electrode. The electrolyte solution contained 0.25 M Na_2_S and 0.35 M Na_2_SO_3_ (pH 13). A 350 W Xe lamp (FL-88) was used as the solar simulated source with AM 1.5 filter (Oriel Instruments Corporation, Stratford, CT, USA, model 81094). The irradiance measurement was detected with a power meter (Newport Opto-Electronics Technologies (Wuxi) Co., Ltd., Jiangsu, China, model 842-PE), and full power irradiation was fixed at 100 mW/cm^2^ throughout this work. A lens (Newport, model LFM-1A) focused light on the working electrode with a surface area of 1 cm^2^. Linear sweep voltammograms ranges were from −0.5 to 0.4 V and the scanning rate was 10 mV/s. By the same method, the photoelectrochemical properties of the CdS nanoparticles-sensitized ZnO thin films without Al-doping and/or hydrogen treatment were measured for comparison. The light switch was tested at a bias of 0 V (vs. Ag/AgCl potential) and 180 s in a cycle with light on and off. The stability of AZO nanorod array thin film with hydrogen treatment was examined by current–voltage (*C*-*V*) scanning from −0.5 to 0.4 V for 1 or 50 cycles at a scanning rate of 10 mV/s under illumination and by measuring the current variation with time under illumination at 0.5 V for 2 h.

## Results and discussion

**Figure 1:**
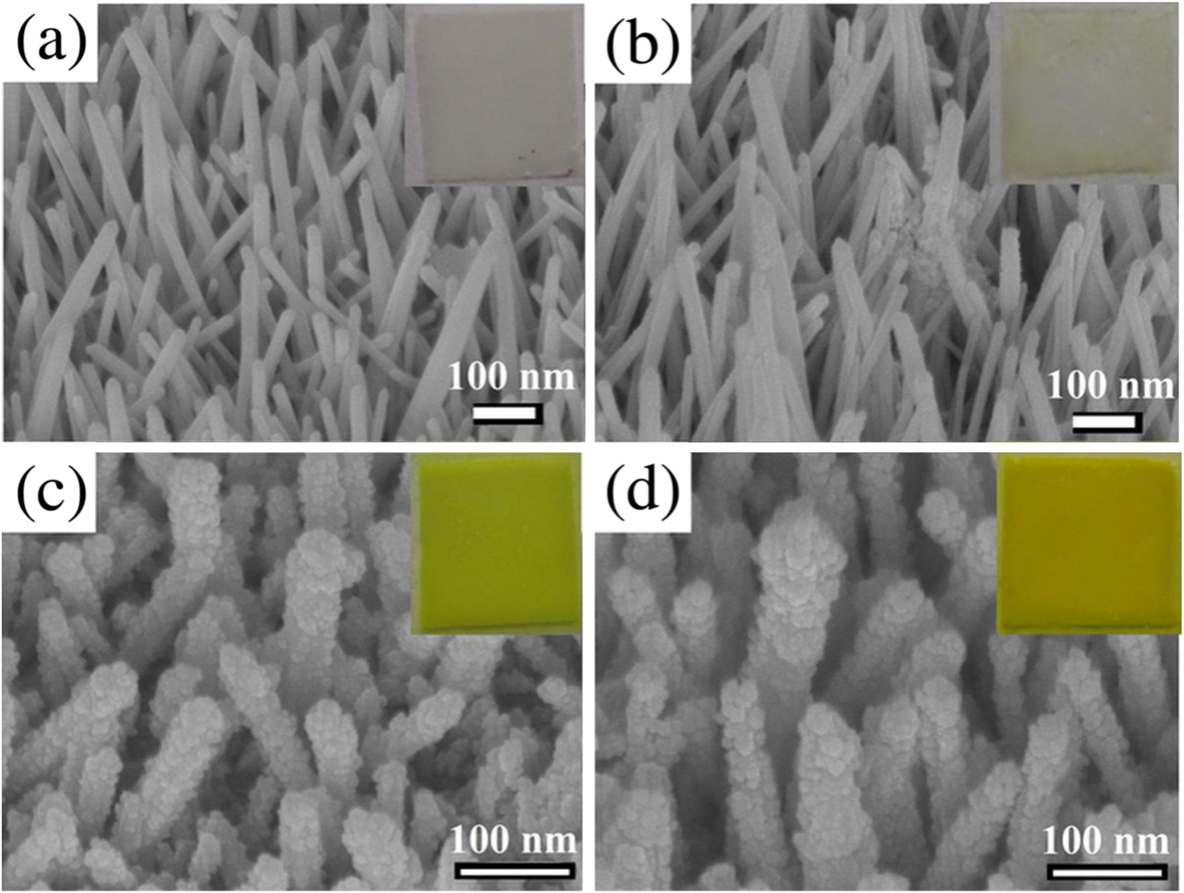
**SEM images of AZO nanorod array thin film.** SEM images of AZO nanorod array thin film with hydrogen treatment (**a**) and those sensitized by CdS nanoparticles for different cycle numbers (**b-d**). Cycle numbers are 1 (b), 3 (c), and 5(d). The insets show the color change for the AZO nanorod array thin films with hydrogen treatment before and after sensitization by CdS nanoparticles.

Figure 1 shows the typical SEM images of AZO nanorod array thin film with hydrogen treatment and those sensitized by CdS nanoparticles for different cycle numbers. It was found that the chemical bath deposition of CdS nanoparticles did not destroy the 1-D morphology of AZO nanorod array thin films. Also, the loading of CdS nanoparticles increased with increasing the cycle number. When the cycle number was 1, the deposition of CdS nanoparticles on the AZO nanorods was incomplete and some larger CdS nanoparticles were observed. From the SEM image, the larger particles were the aggregates of smaller CdS nanoparticles. So their formation might be due to the inhomogeneous deposition of CdS nanoparticles from bulk reaction. When the cycle numbers were 3 and 5, AZO nanorods were deposited by CdS nanoparticles uniformly and completely. No significant aggregation was observed. This might be because the decorated CdS nanoparticles from the first sensitization could act as the seeds for the deposition of more CdS, which reduced the formation of CdS nanoparticles from bulk reaction and led to the more homogeneous deposition of CdS nanoparticles. As for the original aggregates, they might be covered by the CdS nanoparticles which are newly deposited or dissolved and re-deposited on the surface of AZO nanorods. In addition, as shown in the insets of Figure 1, it was observed clearly that the colors of AZO nanorod array thin films gradually changed from white to yellow with the increasing cycle number, reflecting the increase in the loading of CdS nanoparticles. This was consistent with the observation of SEM images.

**Figure 2:**
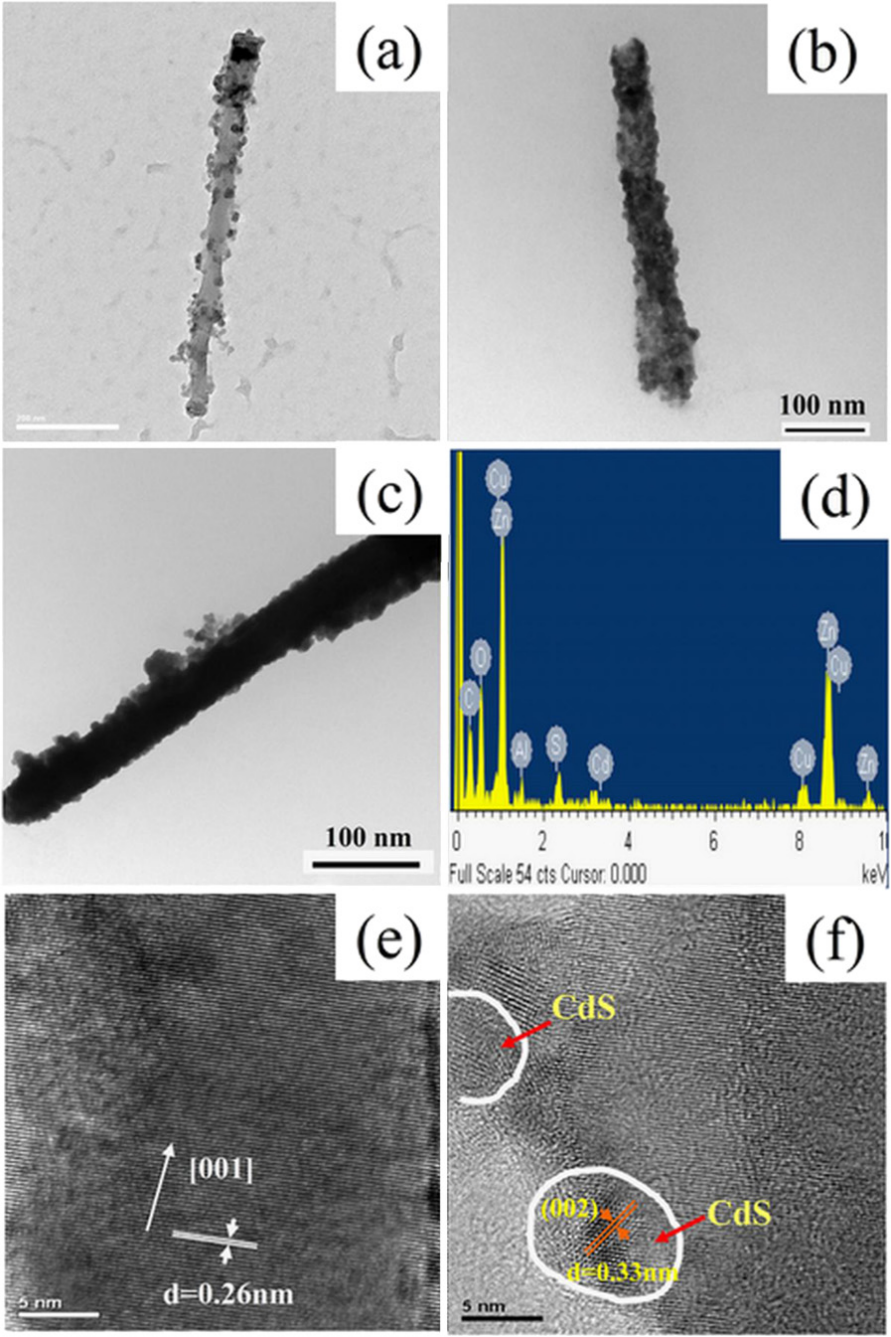
**TEM analysis of the microstructure properties of AZO nanorod array thin film with hydrogen treatment.** TEM images (**a-c**) and EDX spectrum (**d**) of a typical CdS nanoparticles-decorated AZO nanorod as well as the HRTEM image focused on the bare surface (**e**) and that focused on the CdS nanoparticles-decorated surface (**f**). The cycle numbers for CdS nanoparticles sensitization are 1 (a, d-f), 3 (b), and 5 (c).

The microstructure properties of the AZO nanorod array thin film with hydrogen treatment after CdS nanoparticles sensitization were further examined by TEM analysis. For the sample with 1 cycle of sensitization, it could be observed clearly that some CdS nanoparticles were decorated on the nanorod surface as shown in Figure 2a. By increasing the cycle number, more CdS nanoparticles could be decorated on the nanorod surface. When the cycle number was 3, the nanorod surface was covered roughly by a monolayer of CdS nanoparticles as indicated in Figure 2b. When the cycle number increased to 5, the layer of CdS nanoparticles which covered the nanorod surface became thick and dense as shown in Figure 2c. The increase in the surface coverage of AZO nanorod by CdS nanoparticles with the increasing cycle number was consistent with the observation in the SEM images. The rough surface with some aggregate particles might be resulted by the peeling from the thin film. In addition, for the sample with 1 cycle of sensitization, its EDX spectrum (Figure 2d) revealed the presence of Cd and S elements. Also, the atomic ratio of Cd/S was found to be 1.0. This result correlated well with the atomic ratio of CdS. Furthermore, as shown in Figure 2e, its HRTEM image focused on the bare surface confirmed that the AZO nanorod had a single crystal wurtzite structure and was grown along the *c*-axis [001] direction with 0.26 nm {001} lattice fringe parallel to the basal plane as observed in our previous work [44]. Also, from the HRTEM image focused on the nanorod surface with decorated CdS nanoparticles as shown in Figure 2f, it could be observed that the decorated CdS nanoparticles had a mean diameter of about 7 nm and a lattice spacing of 0.33 nm which was related to the (002) plane of hexagonal structure.

**Figure 3:**
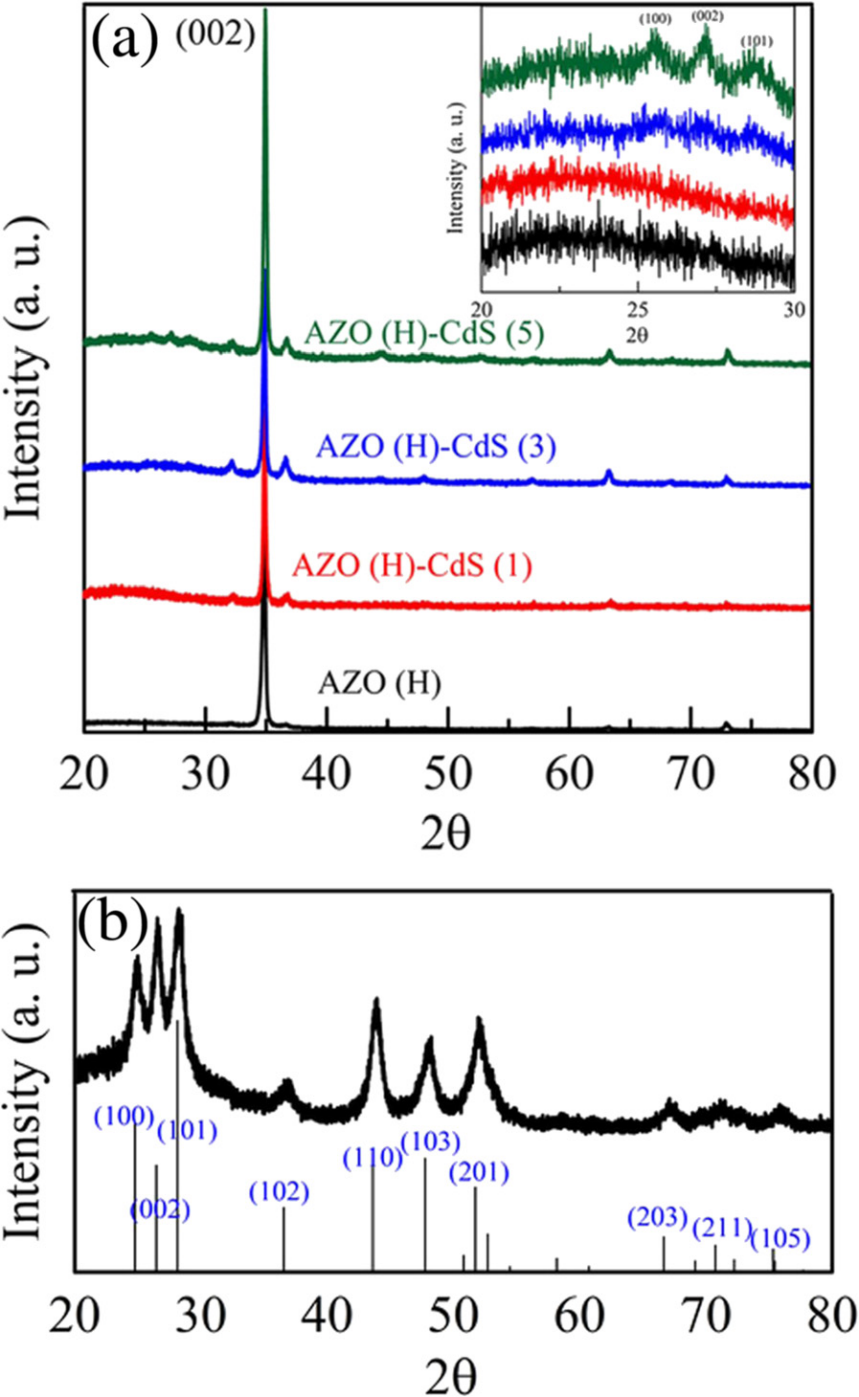
**XRD patterns of AZO nanorod array thin film and CdS nanoparticles.** (**a**) XRD patterns of AZO nanorod array thin film with hydrogen treatment and those after sensitization by CdS nanoparticles for different cycle numbers. (**b**) XRD pattern of CdS nanoparticles synthesized in the absence of AZO nanorod array thin film.

Figure 3a shows the XRD patterns of AZO nanorod array thin film with hydrogen treatment and those after sensitization by CdS nanoparticles for various cycle numbers, in which AZO(H), AZO(H)-CdS (1), AZO(H)-CdS (3), and AZO(H)-CdS (5) denote the AZO nanorod array thin film with hydrogen treatment and those after sensitization by CdS nanoparticles for 1, 3, and 5 cycles, respectively. They all exhibited the strong characteristic peak for the (002) plane of wurtzite-type ZnO (hexagonal) around the scattering angle of 35° as observed in our previous work [44]. In addition, as shown in the inset of Figure 3a, some peaks in the range of 2θ = 20°-30° become more significant with increasing the cycle number of CdS nanoparticles sensitization. They could be attributed to the (100), (002), and (101) planes of hexagonal CdS [46], confirming the decoration of CdS nanoparticles on AZO nanorods. These peaks were not very significant but still visual. This was due to the relatively stronger peak intensity of AZO nanorod array thin film. Similar phenomenon was also found in other work [22]. In addition, the XRD pattern of CdS nanoparticles synthesized in the absence of AZO nanorod array thin film was also shown in Figure 3b for comparison. The characteristic peaks related to the (100), (002), (101), (102), (110), (103), (201), (203), (211), and (105) planes of hexagonal CdS could be observed clearly [46]. This confirmed the formation of CdS nanoparticles.

**Figure 4:**
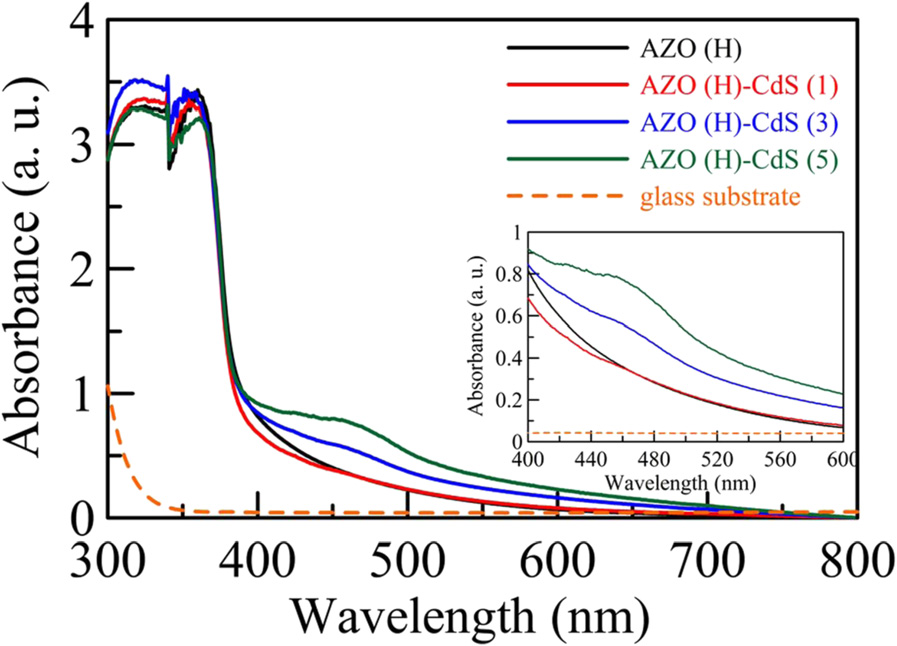
**UV–VIS spectra of AZO nanorod array thin film.** UV–VIS spectra of AZO nanorod array thin film with hydrogen treatment and those sensitized by CdS nanoparticles for different cycle numbers. The UV–VIS spectrum of glass substrate was also shown for comparison. The inset indicates the UV–VIS spectra in the visible light region.

The UV–VIS spectra of AZO nanorod array thin film with hydrogen treatment and those after sensitization by CdS nanoparticles for various cycle numbers were shown in Figure 4, in which the UV–VIS spectrum of glass substrate was also shown for comparison. It was obvious that the glass substrate was transparent above 350 nm, and the absorption of AZO nanorod array thin film with hydrogen treatment occurred mainly in the UV region. After sensitization by CdS nanoparticles, the absorption in the visible light region could be observed. As indicated in Figure 4 and its inset, the characteristic absorption around 460 nm increased with increasing the cycle number. This could be referred to the decoration of CdS nanoparticles on the surface of AZO nanorods. Furthermore, it was noted that the sensitization by CdS nanoparticles for 1 cycle resulted in the slight decrease in the absorption in the range of 375–450 nm. This might be due to that the AZO nanorod array thin film was slightly destroyed during the chemical bath deposition process. Such a phenomenon was also observed in other similar works [21,23]. However, as shown in Figure 1, the 1-D morphology of AZO nanorod array thin films was still retained, and increasing the cycle number did not result in more significant destruction. The XRD analysis as indicated in Figure 3 also revealed that the resulting thin films were mainly composed of AZO after sensitization by CdS nanoparticles for 1–5 cycles. Thus, the chemical bath deposition process for the CdS nanoparticles sensitization should be practicable. In addition, with increasing the cycle number, it was noted that the absorption peak of CdS nanoparticles underwent a slight red-shift with broadening which revealed the growth and broad size distribution of the decorated CdS nanoparticles [24,47]. When the cycle numbers were 1, 3, and 5, the absorption peaks were located at 440, 457, and 470 nm, respectively. The corresponding average sizes of CdS nanoparticles were estimated at 5.1, 5.9, and 6.5 nm, respectively, using the following empirical equation [24,48]:

(1) $$D=\left(-6.6521\times 10^{-8}\right)\lambda^{3}+\left(1.9557\times 10^{-4}\right)\lambda^{2}-\left(9.2352\times 10^{-2}\right)\lambda +13.29\text{,}$$

where *D* (nanometer) was the size of CdS nanoparticles and *λ* (nanometer) was the wavelength of the first excitonic absorption peak. This result was roughly consistent with the observation from the TEM images as indicated in Figure 2.

**Figure 5:**
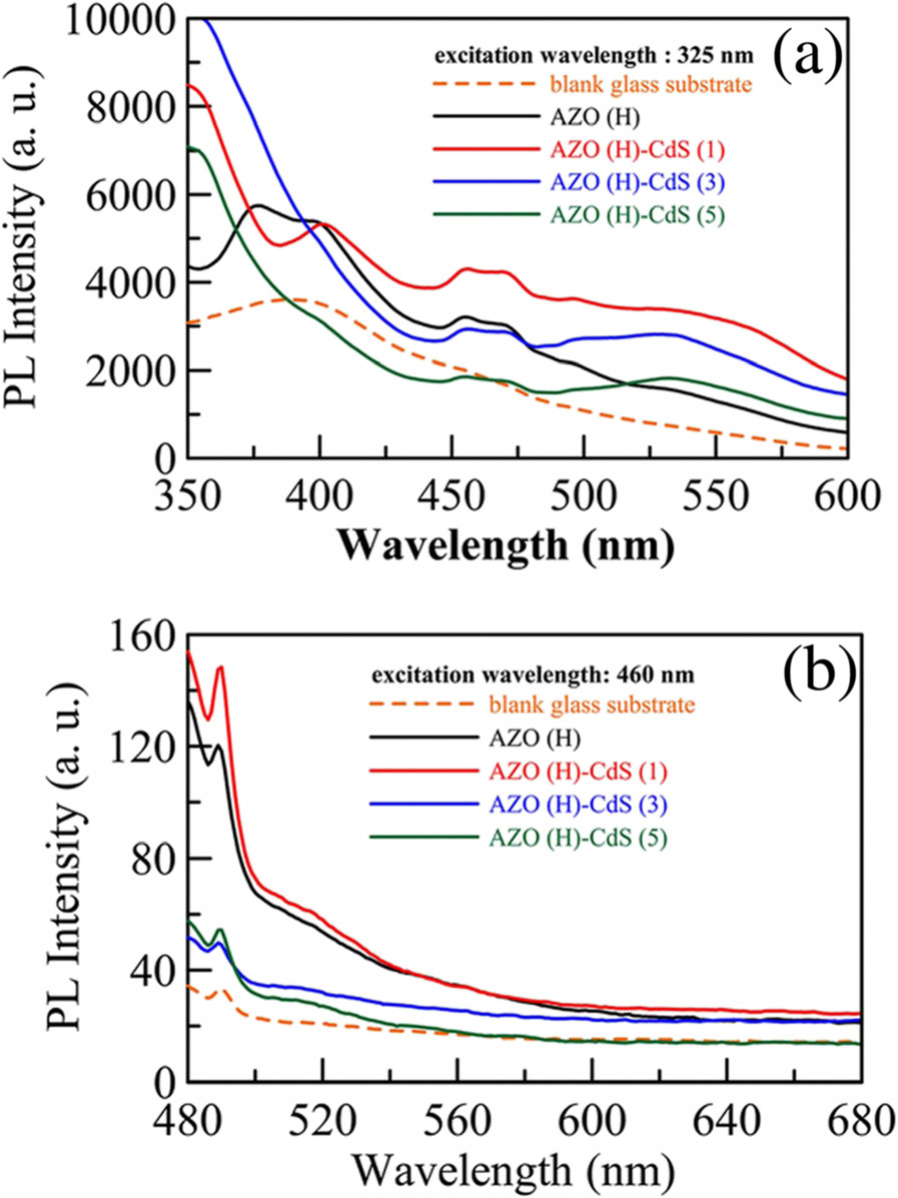
**Photoluminescence spectra of glass substrate as well as the AZO nanorod array thin film.** Photoluminescence spectra of glass substrate as well as the AZO nanorod array thin film with hydrogen treatment and those sensitized by CdS nanoparticles for different cycle numbers. The excitation wavelengths were 325 (**a**) and 460 (**b**) nm.

Figure 5a shows the photoluminescence spectra of AZO nanorod array thin film with hydrogen treatment and those after sensitization by CdS nanoparticles for various cycle numbers at an excitation wavelength of 325 nm. The photoluminescence spectrum of AZO nanorod array thin film with hydrogen treatment displayed an emission peak around 380 nm owing to the band-to-band emission as well as the other emission peak around 465 nm resulting from the surface or defect states [49-51]. With increasing the cycle number, the band-to-band emission peak around 380 nm disappeared gradually. Because the emission of AZO nanorods overlapped with the absorption of CdS nanoparticles, the electron–hole recombination of AZO nanorods might be reduced by CdS nanoparticles by the fluorescence resonance energy transfer (FRET) via the Z-scheme mechanism, in which the electrons in the AZO conduction band could transfer to the CdS valence band and thus quenched the photoluminescence intensity of AZO [25]. Furthermore, after sensitization by CdS nanoparticles, a new broad emission peak around 540 nm appeared and its intensity increased gradually with increasing the cycle number. Because the AZO nanorods had little emission at 540 nm but CdS nanoparticles always exhibit a broad emission due to trap states after the bandgap emission, the new broad emission band around 540 nm could be referred to the trap state emission of the decorated CdS nanoparticles. This confirmed the decoration of CdS nanoparticles on the AZO nanorods. In addition, it was noted that the emission peak around 465 nm also decreased gradually, revealing that the quality of AZO surface could be improved by the sensitization of CdS nanoparticles [51]. This led to the less interfacial charge recombination and was helpful to improve the overall photoelectrochemical performance [51].

The photoluminescence spectra at an excitation wavelength of 460 nm, at which CdS nanoparticles exhibited significant absorption, were also examined. As shown in Figure 5b, a broad emission occurred in the wavelength of 480–600 nm was observed for AZO nanorod array thin film with hydrogen treatment. Because the excitation at 460 nm cannot result in the band-to-band emission of AZO, the emission observed should be resulted from the surface or defect states [49-51]. After sensitization by CdS nanoparticles for 3 and 5 cycles, significant quenching in photoluminescence intensity was observed. As stated above, this was because the sensitization of CdS nanoparticles improved the quality of AZO surface and reduced the interfacial charge-recombination [51].

**Figure 6:**
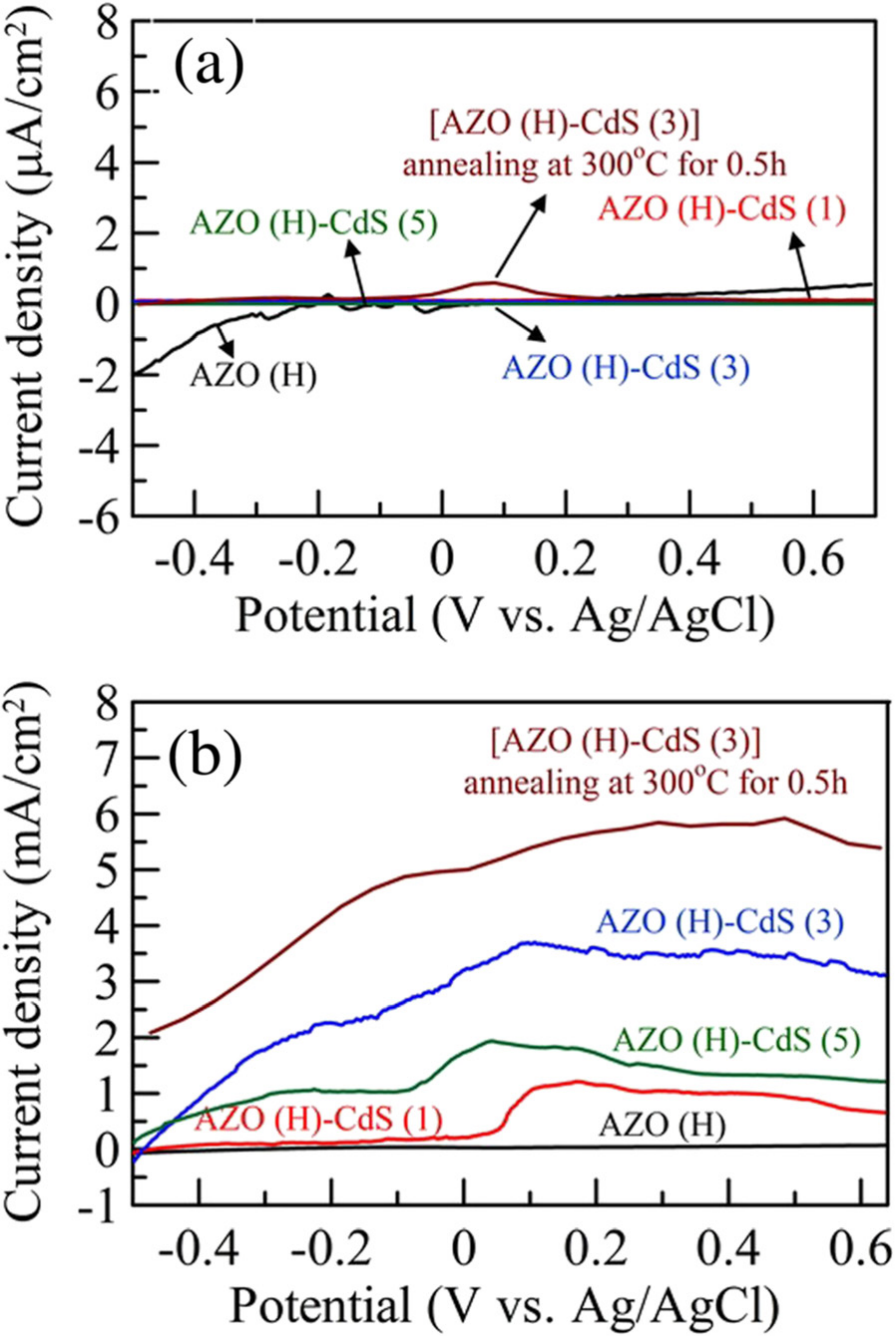
**Linear sweep voltammograms of AZO nanorod array thin film.** Linear sweep voltammograms of AZO nanorod array thin film with hydrogen treatment and those sensitized by CdS nanoparticles for different cycle numbers in the dark (**a**) and under illumination (**b**).

Figure 6 shows the linear sweep voltammograms of AZO nanorod array thin film with hydrogen treatment and those sensitized by CdS nanoparticles for different cycle numbers in the dark and under illumination. As shown in Figure 6a, it was obvious that the dark current densities for all samples were quite low (in the range of 10^−7^ A/cm^2^) and negligible. Under illumination, as indicated in Figure 6b, the AZO nanorod array thin film with hydrogen treatment still showed quite low photocurrent density because of its poor visible light absorption. However, they showed pronounced photocurrent densities after sensitization by CdS nanoparticles. The enhancement effect could be reasonably attributed to the high absorption of CdS nanoparticles in the visible light region [21,22,26]. When CdS nanoparticles absorbed the photons in the visible light region, the excited photoelectrons could be transferred to the conduction band of AZO rapidly so that the charge separation of electron–hole pairs became easier and thereby enhanced the photocurrent [21,26,51]. In addition, for AZO(H)-CdS (1), AZO(H)-CdS (3), and AZO(H)-CdS (5), their photocurrents all started at about −0.5 V, continued to increase, and then roughly approached to the saturation at more positive potentials. The onset potential for AZO(H)-CdS (3) could be evaluated as −0.48 V. Similar phenomenon was also observed in the previous reports [21,22,26]. This revealed that the charge separation has reached the maximum at sufficiently positive potentials [16]. As for the slight decrease of photocurrent for AZO(H)-CdS (5) when the potential was above 0.2 V, it might be due to the instability of excess CdS nanoparticles at a higher positive potential. This also revealed that the appropriate cycle number for CdS nanoparticles sensitization was 3.

Furthermore, as shown in Figure 6, the maximum photocurrent densities were about 1.1, 3.6, and 1.8 mA/cm^2^ for AZO(H)-CdS (1), AZO(H)-CdS (3), and AZO(H)-CdS (5), respectively. Also, the short current densities (i.e., the current at a zero bias potential) could be determined at 0.03, 0.215, 3.21, and 1.73 mA/cm^2^ for AZO(H), AZO(H)-CdS (1), AZO(H)-CdS (3), and AZO(H)-CdS (5), respectively. It was noteworthy that the enhancement increased and then decreased with the increasing cycle number. For AZO(H)-CdS (1) and AZO(H)-CdS (3), it was reasonable that more cycle numbers could result in a higher photocurrent density owing to the increased surface coverage of AZO nanorods by CdS nanoparticles as observed in their SEM images (Figure 1). As for the AZO(H)-CdS (5), its lower enhancement than AZO(H)-CdS (3) might be due to the deposition of excess CdS nanoparticles. Excessive CdS sensitization might cause the increase in the distance for the electron transportation of outer CdS nanoparticles to AZO surface, and also increase the interaction probability of CdS-CdS nanoparticles which might act as the trap or recombination centers of electron–hole pairs [51]. Thus, it was suggested that the monolayer deposition of CdS nanoparticles on the surface of AZO nanorods could produce a maximum photocurrent. This result was in good agreement with the earlier works on the dye [52] or quantum dots-sensitized [53-55] solar cells. Moreover, it was mentionable that the photocurrent density for AZO(H)-CdS (3) was comparable and even slightly superior to some earlier works for the CdS-sensitized ZnO nanorod array thin films with ITO, FTO, or metallic Ti foil as substrates [21,22,26,56]. Thus, the AZO nanorod array thin film with hydrogen treatment indeed could be used as an ITO/FTO-free photoanode, and its performance for solar water splitting could be significantly improved by the sensitization with the quantum dots capable of visible light absorption.

**Figure 7:**
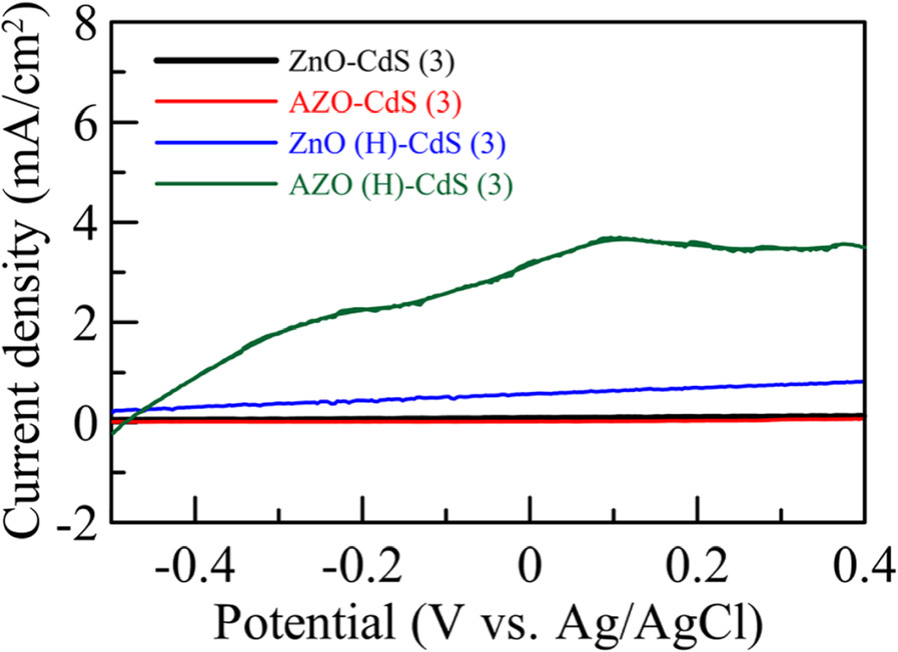
**Linear sweep voltammograms of ZnO and AZO nanorod array thin films with and without hydrogen treatment.** Linear sweep voltammograms of the ZnO and AZO nanorod array thin films with and without hydrogen treatment after sensitization by CdS nanoparticles for 3 cycles under illumination.

As stated above, our recent work revealed that Al-doping and hydrogen treatment could significantly enhance the conductivity of ZnO nanorod array thin film [44]. For comparison, the linear sweep voltammograms of the ZnO and AZO nanorod array thin films with and without hydrogen treatment after sensitization by CdS nanoparticles for 3 cycles were also measured under illumination as indicated in Figure 7 in which ZnO-CdS (3) and AZO-CdS (3) denote the ZnO and AZO nanorod array thin films without hydrogen treatment after sensitization by CdS nanoparticles for 3 cycles. It was found that the ZnO and AZO nanorod array thin films without hydrogen treatment had quite low photocurrent density. Also, for the nanorod array thin films with hydrogen treatment, the photocurrent density of AZO was significantly higher than that of ZnO. This revealed that both the effects of Al-doping and hydrogen treatment were also helpful for the enhancement of photocurrent density, and the enhancement by hydrogen treatment was more significant that that by Al-doping. This phenomenon could be attributed to the enhancement in the conductivity as observed in our previous work [44]. When CdS nanoparticles absorbed the photons in the visible light region, the excited photoelectrons were transferred to the conduction band of AZO. The superior conductivity was helpful for the rapid transfer of electrons within the nanorods, leading to the more efficient electron collection. This demonstrated that the AZO nanorod array thin film with hydrogen treatment possessed significantly better photoelectrochemical property than the ZnO nanorod array thin films without Al-doping and/or hydrogen treatment.

**Figure 8:**
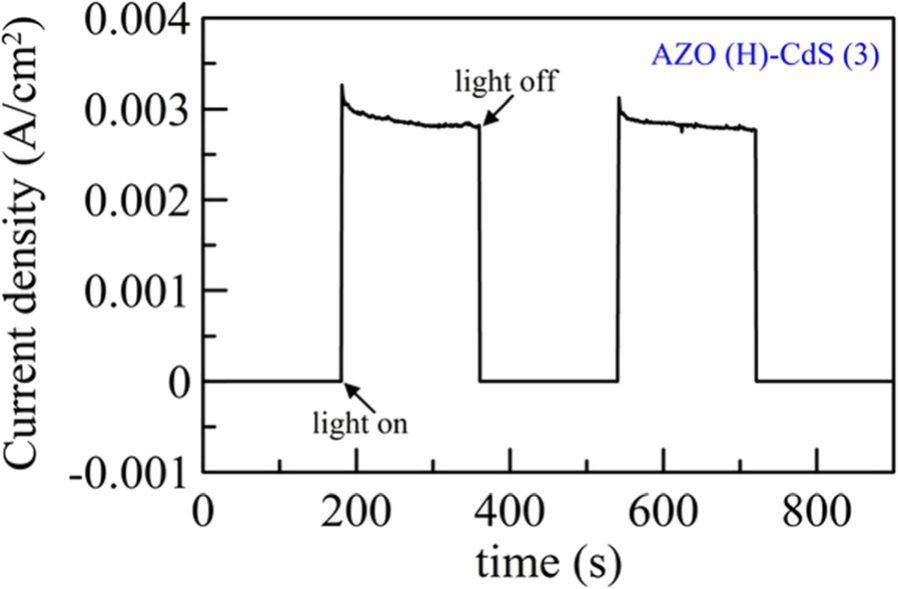
Photocurrent transient behavior of AZO(H)-CdS (3) at a bias of 0 V.

**Figure 9:**
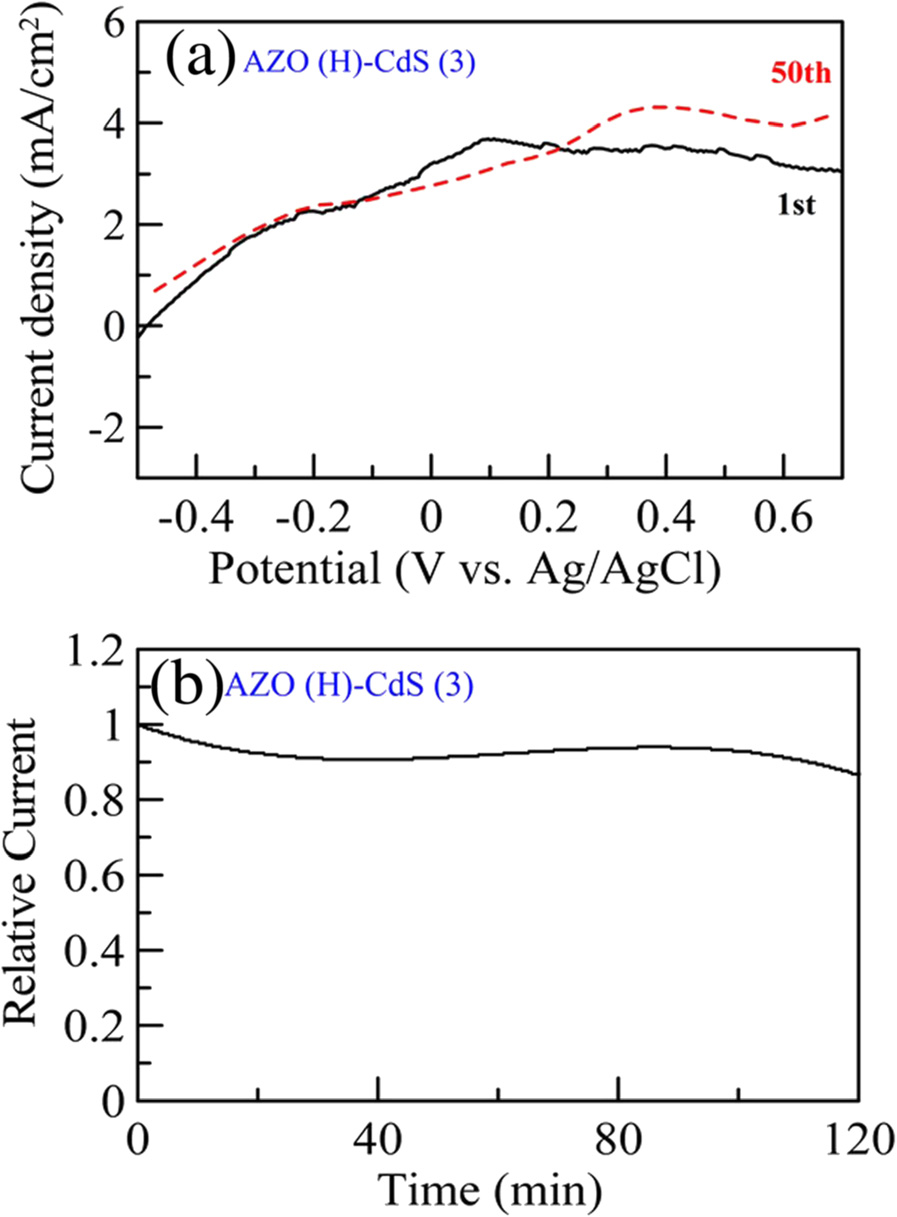
**Linear sweep voltammograms of AZO(H)-CdS (3) after *C-V *scanning and variation of relative current with time.** (**a**) Linear sweep voltammograms of AZO(H)-CdS (3) after *C-V* scanning for 1 and 50 cycles under illumination. (**b**) Variation of relative current with time for AZO(H)-CdS (3) under illumination at 0.5 V.

In addition, Figure 8 shows the photocurrent transient behavior of AZO(H)-CdS (3) at a bias of 0 V with a switch time of 180 s. It indicated that the AZO(H)-CdS (3) had good photosensitivity and reproducibility. In addition, the linear sweep voltammograms of AZO(H)-CdS (3) were measured after *C*-*V* scanning for 1 and 50 cycles under illumination. The result was shown in Figure 9a. It was found obviously that under illumination AZO(H)-CdS (3), after *C-V* scanning for 50 cycles, still showed similar photoelectrochemical property as that after *C*-*V* scanning for 1 cycle. Furthermore, Figure 9b indicates the variation of relative current with time for AZO(H)-CdS (3) under illumination at 0.5 V. No significant current decay was observed in 2 h. Both results revealed that the AZO(H)-CdS (3) had good stability. Accordingly, the AZO nanorod array thin film with hydrogen treatment was indeed suitable as an ITO/FTO-free photoanode for solar water splitting after sensitization by CdS nanoparticles.

**Figure 10:**
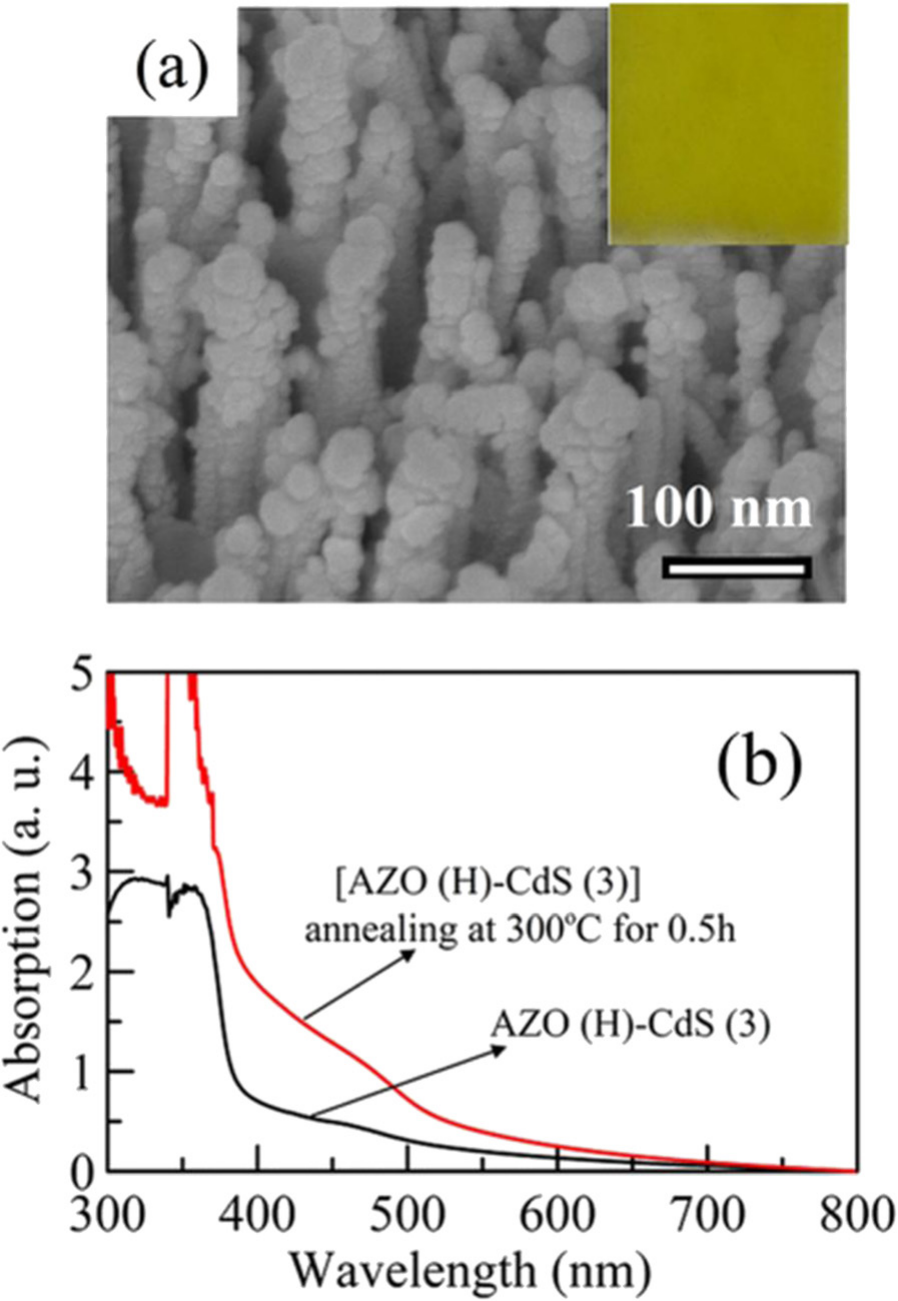
**SEM image and UV–VIS spectra of AZO(H)-CdS (3) before and after annealing at 300°C for 0.5 h.** (**a**) SEM image of AZO(H)-CdS (3) after annealing at 300°C for 0.5 h. The inset shows the color of thin film. (**b**) UV–VIS spectra of AZO(H)-CdS (3) before and after annealing at 300°C for 0.5 h.

Usually, heat treatment could improve the crystallinity and thereby enhance the electron transportation [57]. So the further annealing of AZO(H)-CdS (3) at 300°C was conducted for 0.5 h. The SEM image and UV–VIS spectrum were shown in Figure 10. It was found that, after annealing, the 1-D morphology of AZO(H)-CdS (3) has no significant change. However, the size of CdS nanoparticles became larger owing to the particle sintering. The sintering of CdS nanoparticles also affected the absorption property. It was obvious that, after annealing, the visible absorption in the range of 400 to 500 nm was enhanced largely and the absorption edge was red-shifted. This might be due to the improved crystallinity and larger size of CdS nanoparticles, respectively. Furthermore, the linear sweep voltammograms of annealed AZO(H)-CdS (3) in the dark and under illumination were also shown in Figure 6. It was obvious that the photocurrent density was enhanced significantly after annealing. A maximum short current density of 5.03 mA/cm^2^ could be obtained under illumination. In addition to the crystallinity improvement, this also might be partially due to the enhanced and red-shifted absorption which raised the utilization of light. This demonstrated that the photoelectrochemical performance of CdS nanoparticles-sensitized AZO nanorod array thin film with hydrogen treatment indeed could be enhanced by heat treatment.

## Conclusions

The AZO nanorod array thin film with hydrogen treatment has been sensitized by CdS nanoparticles successfully via chemical bath deposition as a novel ITO/FTO-free composite photoelectrode for solar water splitting. The sensitization not only did not destroy the 1-D morphology of AZO nanorod array thin film, but also could efficiently increase the absorption around 460 nm and reduce the electron–hole recombination of AZO nanorods via the FRET mechanism. The CdS nanoparticles decorated on AZO nanorods had a hexagonal structure and the diameters of 5.1-6.5 nm. By increasing the cycle number, the loading of CdS nanoparticles was raised and could significantly enhance the photoelectrochemical property of AZO nanorod array thin film with hydrogen treatment. When a monolayer of CdS nanoparticles was formed on AZO nanorods, the maximum short current density under illumination could be obtained as 3.21 mA/cm^2^ which was much higher than those without CdS nanoparticles sensitization and those with CdS nanoparticles sensitization but without Al-doping and/or hydrogen treatment. Such a good performance was comparable and even slightly superior to some earlier works for the CdS-sensitized ZnO nanorod array thin films with ITO, FTO, or metallic Ti foil as substrates. In addition, the CdS nanoparticles-decorated AZO nanorod array thin film with hydrogen treatment also exhibited good photosensitivity, reproducibility, and stability. After further annealing at 300°C for 0.5 h, the maximum short current density under illumination could be raised to 5.03 mA/cm^2^. Accordingly, we successfully demonstrated that the AZO nanorod array thin film with hydrogen treatment could be used as a novel ITO/FTO-free photoanode, and its performance for solar water splitting could be significantly improved by CdS nanoparticles sensitization.

## Competing interests

The authors declare that they have no competing interests.

## Authors’ contributions

Both authors read and approved the final manuscript.
